# Supplementary material for: The interactome of intact mitochondria by cross-linking mass spectrometry provides evidence for coexisting respiratory supercomplexes
Source: Mol Cell Proteomics. 2017 Dec 8;17(2):216–32. doi: 10.1074/mcp.RA117.000470 (PMC5795388; doi:10.1074/mcp.RA117.000470)
Supplement: Supplemental Data [file supp_RA117.000470_133922_0_supp_23978_fzffwf.zip › spectra_annotation/mito_DR_spectra_annotation/391-1-14-1-4-1.pdf]

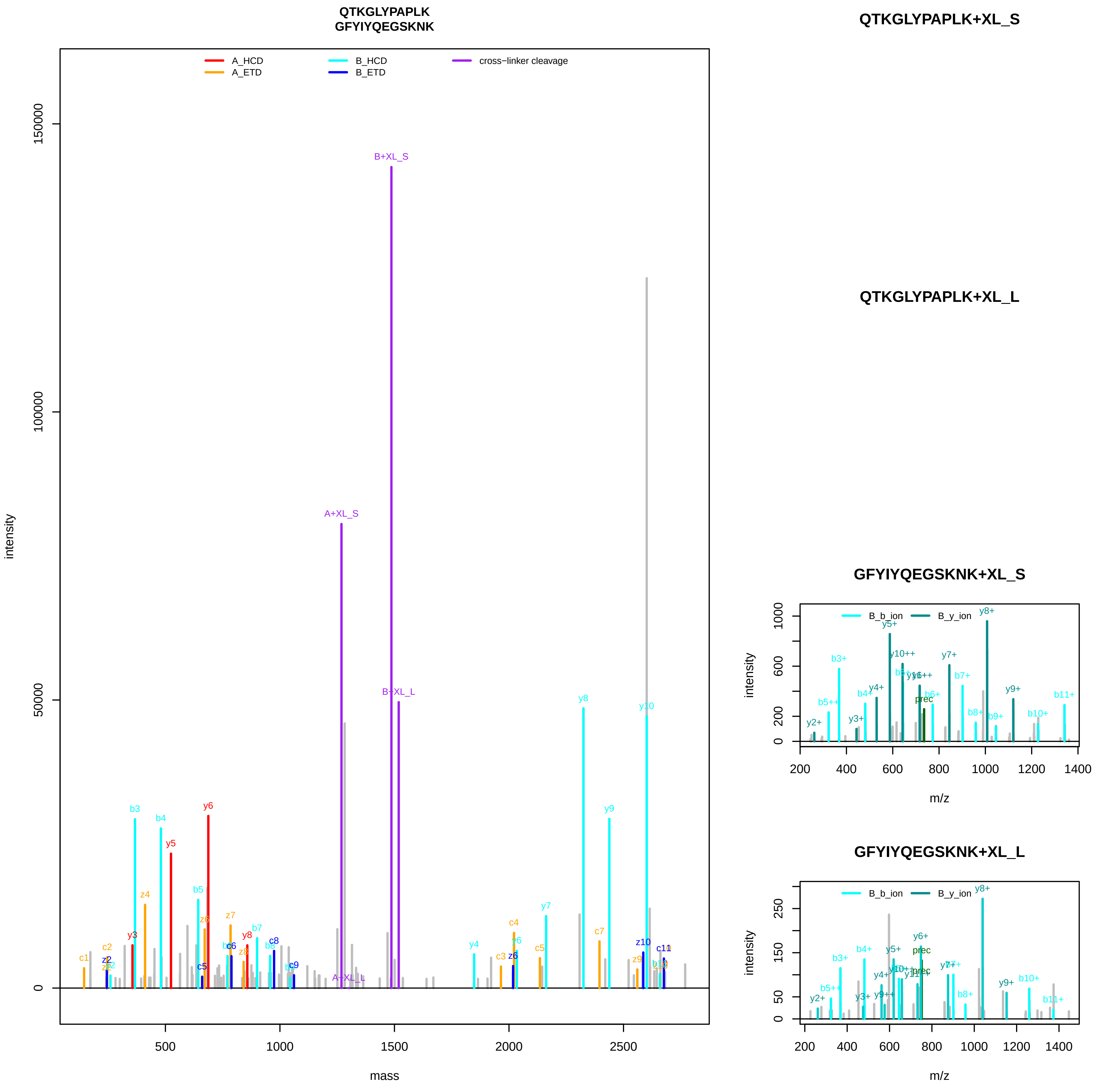
QTKGLYPAPLK
GFYIYQEGSKNK
A_HCD
A_ETD
B_HCD
B_ETD
cross−linker cleavage
intensity
mass
0
50000
100000
150000
500
1000
1500
2000
2500
B+XL_S
A+XL_S
B+XL_L
A+XL_L
y8
y10
y9
y7
y6
y4
c4
c3
z6
c5
c7
z9
z10
c11
b3
b4
y6
y5
b5
z4
y3
z7
y8
c1
c2
c5
c6
c8
c9
b7
QTKGLYPAPLK+XL_S
QTKGLYPAPLK+XL_L
GFYIYQEGSKNK+XL_S
B_b_ion
B_y_ion
y8+
y5+
y10++
b3+
y7+
b7+
b6+
b5++
b4+
y4+
y3+
y2+
prec
b8+
b9+
y9+
b10+
b11+
0
200
600
1000
m/z
200
400
600
800
1000
1200
1400
GFYIYQEGSKNK+XL_L
B_b_ion
B_y_ion
y8+
y6+
prec
b3+
b4+
y5+
b5++
y2+
y3+
y9++
y4+
b8+
y9+
b10+
b11+
0
50
150
250
